# 12-item version of Boston Naming Test: usefulness in the diagnosis of primary progressive aphasia, frontotemporal dementia, and Alzheimer's disease

**DOI:** 10.1590/1980-5764-DN-2021-0043

**Published:** 2022-04-29

**Authors:** Héctor Gastón Graviotto, Marcos German Sorbara, Carlos Mario Turizo Rodriguez, Cecilia Serrano

**Affiliations:** 1 Unidad Asistencial Dr César Milstein, Department of Neurology – Buenos Aires, Argentina.

**Keywords:** Aphasia, Anomia, Language Tests, Dementia, Afasia, Anomia, Testes de Linguagem, Demência

## Abstract

The 12-item version of the Boston Naming Test (BNT) was adapted to Argentina for the detection of dementia due to Alzheimer's disease (AD), with scores similar to the original 60-item version (sensitivity and specificity of 85 and 94%, respectively) without demographic influence (age and educational level). To date, no publications on the use of abbreviated BNT in other degenerative pathologies with language impairment have been reported.

**Objective:**

The objective of this study was to evaluate the usefulness of 12-item BNT in primary progressive aphasia (PPA), the behavioral variant of frontotemporal dementia (FTDbv), and AD.

**Methods:**

Notably, 47 patients with probable AD (NIA-AA 2011) — clinical dementia rating (CDR) 0.5–1, 55 with FTDbv, 17 with PPA, and 46 controls were evaluated and matched for age and education. Exclusion criteria were as follows: alcoholism, other previous neurological or psychiatric illnesses, and education <4 years. All were assessed with a full neuropsychological battery and a 12-item version of BNT.

**Results:**

Median scores of 12-item BNT were as follows: PPA: 3.87 (SD=2.99), AD: 6.13 (SD=3.03); FTDbv: 8.41 (SD=2.53); and controls: 10.22 (SD=1.82). Receiver Operating Characteristic (ROC) curves were plotted.

**Conclusions:**

The 12-item version of BNT can be useful, simple, and fast to identify and differentiate PPA, FTDbv, and AD from controls while retaining the discriminative ability of the original version.

## RESUMO.

A versão de 12 itens do Teste de Nomeação de Boston (TNB) foi adaptada para a Argentina para a detecção de demência por doença de Alzheimer (DA), com escores semelhantes à versão original de 60 itens (sensibilidade e especificidade de 85 e 94%, respectivamente) sem influência demográfica (idade e escolaridade). Até o momento, não foram relatadas publicações sobre o uso do TNB abreviado em outras patologias degenerativas com comprometimento da linguagem.

**Objetivo:**

avaliar a utilidade do TNB de 12 itens na afasia progressiva primária (APP), na variante comportamental da demência frontotemporal (DFT) e na doença de Alzheimer (DA).

**Métodos:**

47 prováveis DA (NIA-AA 2011) — CDR 0,5–1, 55 DFT, 17 APP e 46 controles foram avaliados e pareados por idade e escolaridade. Critérios de exclusão: alcoolismo, outras doenças neurológicas ou psiquiátricas prévias e escolaridade <4 anos. Todos foram avaliados com uma bateria neuropsicológica completa e versão de 12 itens do TNB.

**Resultados:**

medianas das pontuações de 12 itens TNB: APP: 3,87 (DP=2,99), DA: 6,13 (DP=3,03); DFT: 8,41 (DP=2,53) e Controles: 10,22 (DP=1,82). As curvas ROC foram traçadas.

**Conclusões:**

O TNB de 12 itens pode ser útil, simples e rápido para identificar e diferenciar APP, DFT e DA nos controles, mantendo a capacidade discriminativa da versão original.

## INTRODUCTION

Aphasia is an acquired language disorder caused by brain damage. It can affect production, understanding, or both. Acute onset cerebrovascular accident is usually the most common cause of aphasia, affecting at least one-third of people, with considerable sequelae until spontaneous recovery of language^1^.

Certain dementias usually manifest with aphasic forms and should be taken into account as a differential diagnosis, although their symptoms and evolution are different.

The two main dementias in which language disorders probably represent an early presenting feature are Alzheimer's type dementia and primary progressive aphasia (PPA) as the second major form of frontotemporal degeneration. In Alzheimer's disease (AD), cognitive impairment extends beyond language and typically affects episodic (i.e., anterograde or day to day) memory. In PPA, the gradual deterioration of language skills is contrasted with the relatively preserved nonverbal skills and daily activities. Progressively, more communication difficulties and greater cognitive impairment appear^2,3^. Occasionally, the behavioral variant of frontotemporal dementia (FTDbv) can begin with a language disorder and is presented simultaneously with executive or behavioral disorders.

The type of aphasia in AD usually depends on the stage of the disease. In the early stages, there may be slight word-finding difficulties, with occasional semantic paraphasia (e.g., semantic substitutions such as saying “aunt” instead of “sister”), but the speech is still fluent and grammatically correct like an anomic aphasia. With disease progression, patients present with transcortical sensory aphasia, in which there is evident anomia and comprehension is severely impaired. In the moderate to severe stages, there is a reduced lexical production, and in the most severe stages, echolalia and verbal stereotypies may be evident^2,3^.

The PPAs are classified as fluent, nonfluent, or logopenic variant^4,5^. In the fluent variant, speech remains fluent, with normal prosody, good articulation, and grammatically correct; it still becomes circumlocution progressively and lacks content. Language impairment is associated with a deficit of semantic memory and is, therefore, often referred to as semantic dementia, as it associates aphasia with early comprehensive compromise with later associative agnosia and behavioral disturbances. In the nonfluent-agrammatic variant, speech is forced, hesitant, and choppy, with phonemic paraphasias (e.g., “prinoceros” instead of “rhinoceros”). In the logopenic variant, speech is characterized by logopenia (fluctuation of verbal fluency), anomias, and noticeable disturbances in the repetition of words and phrases^6,7^.

Localized atrophy in the frontotemporal lobes in FTDbv often involves language-related brain networks, suggesting that FTDbv may lead to language dysfunction, especially when processing verbal associations, searching the verbal lexicon, or planning propositional utterances is required.

Therefore, word-finding difficulty or anomia is one of the basic disorders observed in aphasias, as well as a clear marker of cortical profile in dementia syndromes and an early neuropsychological sign of AD^6–8^.

The most widely used way to assess naming is the Boston Naming Test (BNT), which consists of 60 object figures, to be named in increasing order of difficulty. Currently, it is an essential test for the study of semantic memory in dementia assessment protocols. In Buenos Aires, a 60-slide version of the BNT was developed, adapted, and standardized for the adult population^9^.

The BNT is of great help in the diagnosis of dementias, but its length has led to the development of shortened versions that maintain the original objectives and criteria^10^. Several abbreviated forms have been proposed. The only Argentine version, which includes the administration of 12 slides instead of 60, was adapted by Serrano et al., in 2001, maintaining the sensitivity and specificity of 85 and 94%, respectively, similar to the version applied to AD^10^.

There are no publications in the literature on the usefulness of the abbreviated Argentine version of the BNT in non-Alzheimer's degenerative pathologies with language involvement. The aim of this study was to evaluate the sensitivity and specificity of the abbreviated version of the BNT in the differential diagnosis of degenerative pathologies with aphasic predominance: PPA, FTDbv, and AD.

## METHODS

### Type of study

Diagnostic test validation study: It is a observational, analytical, retrospective, cross-sectional, and case-control study.

### Participants

The sample consists of adult subjects, who consulted for cognitive disorders during the period of 2014–2017 in the neurology service of the César Milstein Hospital. The inclusion criteria were to have ≥4 years of schooling and no history of alcoholism and other neurological diseases, such as stroke, severe cerebrovascular disease by neuroimaging, Parkinson's disease, severe traumatic brain injuries (with loss of consciousness, contusions, or hematomas), multiple sclerosis, primary epilepsy, neuromuscular diseases, or previous psychiatric diseases (e.g., major depression, bipolar, and schizophrenia).

### Instrument

The 12-item BNT is a brief naming test. It consists of the following figures: helicopter, octopus, mask, volcano, harmonica, stilt, domino, cactus, hammock, pyramid, muzzle, and palette.

### Methodology

The sample was evaluated by means of the 12-item BNT and a complete cognitive evaluation that included the following tests: Signoret Verbal Memory Test, Boston Naming Test (BNT), Spanish version, Verbal Fluency Test, Trail making test A and B, Digit span, Clock Test, Clinical dementia rating (CDR) test, and The Lawton Instrumental Activities of Daily Living Scale. The neuropsychological protocol and the abbreviated version of the BNT were administered on different days and by two different evaluators, who were blinded to the results obtained by each one of them.

Subjects were evaluated with a neurological and neuroimaging examination (e.g., magnetic resonance imaging [MRI] or computed tomography [CT]) according to the neurology service's protocol for the study of cognitive disorders.

Subsequently, the participants were classified by the type of pathology into the following groups: probable AD according to the criteria of NINCDS-ADRDA^11^ (very mild stage: CDR 0.5–1), behavioral variant of FTDbv according to the criteria of Rascovsky et al.^12^, and PPA according to the criteria of Gorno-Tempini et al.^13^ Highly functional patients were selected (≥6) according to the Lawton Instrumental Activities of Daily Living (score ranges from 0=low function to 8=high function). A control group of subjects without cognitive complaints and with a normal cognitive evaluation (usually corresponding to relatives, close friends, or caregivers of the patients) was also selected.

### Statistical analysis

Demographic differences between the different groups were evaluated using an analysis of variance (ANOVA) test and Student's t-test, as appropriate. Performance on the abbreviated BNT was assessed between the different groups by means of an ANOVA. To analyze the correlation of the test with age and education, the Spearman correlation coefficient was used, and Receiver Operating Characteristic (ROC) curves were drawn to analyze the discriminative capacity of the test in each of the subgroups. A significance level of 5% was used to reject the null hypothesis. The statistical analysis was processed using the statistical package SPSS version 25 (IBM corporation).

### Ethical analysis

To guarantee the ethical aspects of the research, the entire project was carried out by following the current National and International Standards including the recommendations of the Declaration of Helsinki of the AMM of 1964 and subsequent amendments; the Belmont Report: “respect for people, charities and Justice”; CIOMS Guidelines: “ethical principles that should govern the execution of research in human beings”; and Law 3,301 in accordance with Basic Health Law No. 153 and Resolution 1480/2011 of the National Ministry of Health.

This study was approved by the Institutional Ethics Committee under the supervision of the Central Committee of the city of Buenos Aires.

## RESULTS

A total of 165 adult subjects were selected and classified by the type of pathology into 4 groups: 47 patients with probable AD (NINCDS-ADRDA, 1984)^11^ — CDR 0.5–1, 55 with FTDbv^12^, 17 with PPA^13^, and 46 were controls (subjects without cognitive impairment with normal cognitive study).

### Demographic comparison by subgroups

The ANOVA test (ANOVA with the Bonferroni correction for multiple comparisons) was used to compare the variables of age and years of schooling between the four subgroups, and no statistically significant differences were found (p>0.05) (Table 1).

**Table 1:** The 12-item version of the BNT: demographic comparison by subgroups.

	AD (n=46)	FTDbv (n=55)	PPA (n=17)	Control (n=47)
Age	68.3 (SD=5.41)	68.5 (SD=8.30)	71.7 (SD=8.06)	70.2 (SD=7.61)
Gender (%male)	34.8	43.6	23.5	34
Years of schooling	92,609 (SD=3.56)	106,909 (SD=4.31)	95,294 (SD=3.20)	9,766 (SD=3.59)
12-Item version of the BNT median score	6.13 (SD=3.03)	8.41 (SD=2.53)	3.87 (SD=2.99)	10.22 (SD=1.82)

BNT: Boston Naming Test; SD: standard deviation.

### Performance of the reduced version of Boston Naming Test

**Figure 1:**
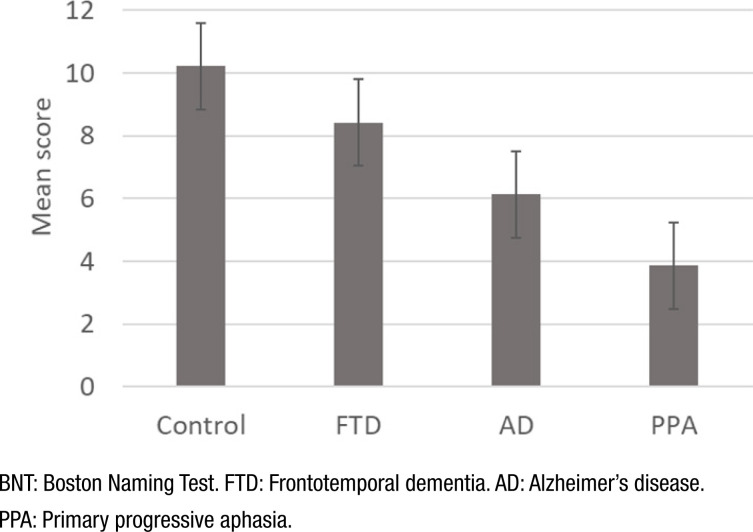
Performance of the 12-item BNT by subgroups.

For the comparison of performance between the subgroups (ANOVA with the Bonferroni correction for multiple comparisons), the difference in performance between all the subgroups was significant (p<0.05), in which worse performance is observed in those patients with a diagnosis of PPA, followed by those with a diagnosis of AD (Figure 1).

### Discriminative capacity of the test

The correlation of the test with age and education was analyzed, and the Spearman correlation coefficient was used.

**Figure 2:**
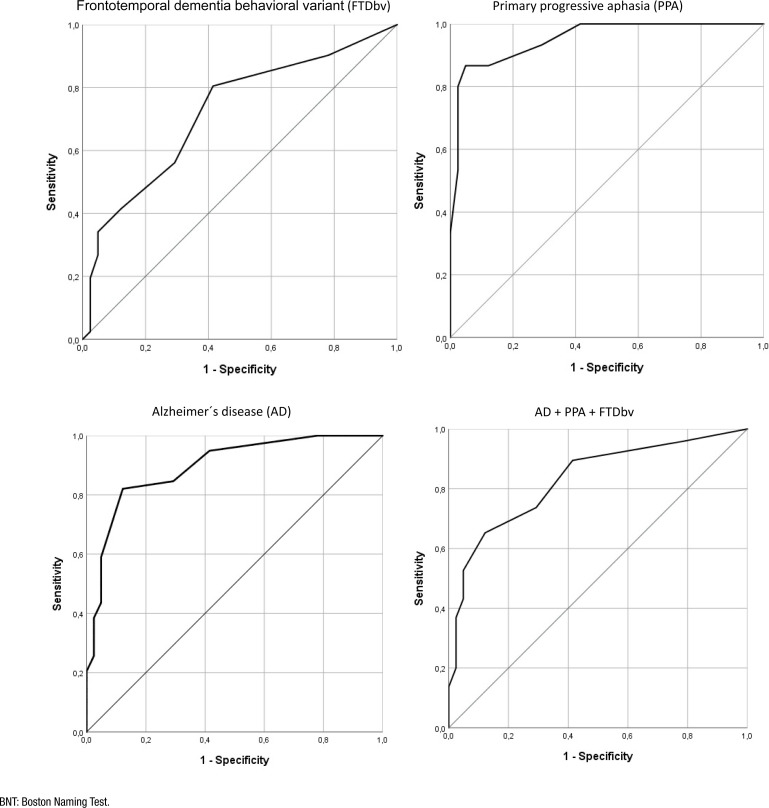
Receiver Operating Characteristic curves of 12-item BNT by subgroups.

The ROC curves for the reduced version of BNT were drawn for PPA vs. controls, for AD vs. controls, and FTDbv vs. controls. The ROC curves are presented in Figure 2, and the area under the ROC curve was of 0.951 (95%CI [0.892–1]) for PPA, 0.895 (95%CI [0.825–0.965]) for AD, and 0.721 (95%CI [0.610–0.832]) for FTDbv.

Thereupon, sensitivity, specificity, and the predictive values (+) and (−) were calculated. The Youden index was applied to establish an optimal cutoff point for pathology discrimination using the abbreviated version of BNT. The sensitivity and specificity of the test were optimized for the population sample by using a cutoff point of 8 (any score of ≤7 was considered as an abnormal result). With this cutoff point, a better balance between sensitivity and specificity was achieved. The sensitivity of the test to detect PPA was 86.7% for PPA, 82.1% for AD, and 41.5% for FTDbv. Specificity was defined as the percentage of controls that scored at or above the cutoff score of 8. The reduced version of BNT had a specificity of 87.8%.

## DISCUSSION

Screening tests for dementia should be widely explored in Latin America^14^. The vast majority of those that exist are intended for the diagnosis of Alzheimer's dementia. Atypical presentations or non-Alzheimer's dementia with aphasic manifestations can offer a great diagnostic challenge. Therefore, validations and adaptations of short batteries for both AD and other types of dementia are of great importance.

Aphasia is often present in several dementias, and its finding is synonymous with pathology^5^. Aphasia arises from disruption of the structural integrity and interconnectivity of the extensive network of the language system. Anomia, at least in spontaneous speech and simple picture naming tasks, could be due to extralinguistic deficits or impairment of the underlying semantic/conceptual system. Extralinguistic impairments may include inattention to the task, forgetting the target word, or being distracted by related competing responses^15^.

All variants of PPA have been shown to have decreased connectivity between inferior frontal gyrus (IFG) and medial temporal gyrus (MTG). The semantic variant generally shows an additional loss of connectivity between the anterior temporal lobe (ATL) and other linguistic regions. In general, the intensity of connectivity in IFG-MTG regions in PPA is correlated with repetition and grammar tasks, whereas MTG-ATL connectivity is associated with picture naming and single-word comprehension.

Altered connectivity in PPA may reflect not only irreversible loss of cortical components due to atrophy but also dysfunction of the remaining neurons.

It is necessary, then, to be able to assess naming, one of the key elements since its alteration is synonymous with aphasia, mainly in PPA, where aphasia is the central diagnosis and in other entities where aphasic disorder is part of the diagnosis (FTDbv and AD)^16^.

Our findings indicate that the abbreviated version of BNT is a simple and fast battery and may be useful to differentiate normal aging from language dysfunction. Specifically, the results suggest that it could be a valid tool to identify PPA, FTDbv, and AD from healthy subjects.

As expected, the best diagnostic performance was observed in patients with PPA; nevertheless, the test showed an excellent performance in patients with Alzheimer's type dementia followed by acceptable results in FTDbv.

The abbreviated version of the BNT, as we described in the initial publication when administered to Alzheimer's subjects, has no demographic influence^10^.

Language dysfunction is a central element in dementia and is not limited to the classic subvariants, has the ability to assess and identify from an early stage, can help an accurate diagnosis of a specific type of disorder, can improve the understanding of these, can modify the prognosis, and can change the direction of treatment^17^.
